# Which Is More Suitable for First‐Line Treatment of Extensive‐Stage Small Cell Lung Cancer, PD‐L1 Inhibitors Versus PD‐1 Inhibitors? A Systematic Review and Network Meta‐Analysis

**DOI:** 10.1111/crj.13804

**Published:** 2024-07-29

**Authors:** Wenjing Liu, Lulin Yu, Yuqian Feng, Siyu Huang, Yuxin Hua, Mingying Peng, Shanming Ruan, Kai Zhang

**Affiliations:** ^1^ Department of Medical Oncology The First Affiliated Hospital of Zhejiang Chinese Medical University Hangzhou China; ^2^ Department of Medical Oncology The First Affiliated Hospital of Zhejiang Chinese Medical University (Zhejiang Provincial Hospital of Chinese Medicine) Hangzhou China; ^3^ Department of Medical Oncology Anji Traditional Chinese Medical Hospital Huzhou Zhejiang China

**Keywords:** efficacy, extensive‐stage small cell lung cancer, first‐line therapy, PD‐1 inhibitors, PD‐L1 inhibitors, safety

## Abstract

**Background:**

In this network meta‐analysis (NMA), the efficiency and safety of PD‐1 inhibitors + chemotherapy and PD‐L1 inhibitors + chemotherapy were compared in the first‐line therapy of patients with extensive‐stage small cell lung cancer (ES‐SCLC).

**Methods:**

We searched research databases, conference abstracts, and trial registries and subsequently chose relevant studies and extracted dates. The NMA was conducted to estimate the efficiency and safety of the PD‐1 inhibitors + chemotherapy and PD‐L1 inhibitors + chemotherapy on overall survival (OS), progression‐free survival (PFS), overall remission rate (ORR), and adverse events (AEs). Studies were assessed for quality. Subgroup analyses were used to evaluate study heterogeneity.

**Results:**

We included six randomized trials with a total of 3163 patients. Direct comparisons showed that patients who received either PD‐1 inhibitors + chemotherapy (HR: 0.71, 95% CI: 0.57–0.87) or PD‐L1 inhibitors + chemotherapy (HR: 0.74, 0.61–0.89) demonstrated significantly longer OS than those who received placebo + chemotherapy. The results of the NMA showed that no significant differences in OS (HR 0.96 95% CI: 0.72–1.3), PFS (HR 0.83, 95% CI: 0.51–1.4), and ORR (OR 1.3 95% CI: 0.66–2.5) were observed for PD‐1 inhibitors + chemotherapy compared with PD‐L1 inhibitors + chemotherapy, but the Bayesian ranking revealed that patients receiving PD‐1 inhibitors + chemotherapy tended to have longer OS, PFS benefit, and better treatment response than patients receiving PD‐L1 inhibitors + chemotherapy. In terms of safety, no significant difference was observed in their safety profiles.

**Conclusion:**

In comparison to placebo + chemotherapy, PD‐L1 inhibitors + chemotherapy and PD‐1 inhibitors + chemotherapy significantly improved survival for ES‐SCLC. According to the available data, PD‐L1 inhibitors + chemotherapy and PD‐1 inhibitors + chemotherapy had equivalent efficacy and safety; however, the level of evidence of this type of comparison is limited.

## Graphical abstract

Identify the optimal combination strategy in first line of patients with ES‐SCLC. There is no significant difference between PD‐1 plus chemotherapy and PD‐L1 plus chemotherapy in the first‐line therapy of patients with ES‐SCLC.

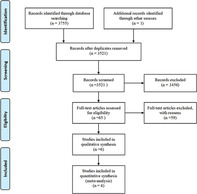

## 1 Introduction

Small cell lung cancer (SCLC), an exceedingly aggressive, poorly diagnosed cancer with high‐grade neuroendocrine features which accounts for up to 15% of all lung cancers, has been labeled a refractory tumor because of its accelerated growth, quick metastasis, and a discouraging 5‐year survival rate (< 5%) [1]. SCLC is divided into two types by the Veterans Affairs Administration Lung Cancer Study Group: limited‐stage SCLC (LS‐SCLC) and ES‐SCLC, with ES‐SCLC being diagnosed in approximately 60% of patients [2, 3, 4, 5]. For the past 30 years, etoposide plus cisplatin or carboplatin has served as the standard first‐line therapy for patients with ES‐SCLC. However, this regimen has only been able to prolong patients' lives by 8–10 months, and while most patients were initially sensitive to chemotherapy, they quickly develop resistance to it. As a result, they are highly susceptible to secondary progression in the short term, with a 5‐year overall survival rate of only about 3% [6, 7, 8]. Therefore, there is an urgent need to improve first‐line treatment of ES‐SCLC.

With the emergence of the immunotherapy era, the biology and clinical characteristics of SCLC offer hopeful prospects for the potential efficacy of immunomodulatory therapy. These include high mutational load, high immunogenicity, and loss of major histocompatibility complex (MHC) molecule expression, which are anticipated to release tumor neoantigens capable of triggering an immune response and serving as the foundation for immunotherapy. Thus immune checkpoint inhibitors (ICIs), represented by PD‐1 inhibitors and PD‐1 inhibitors, have changed the therapeutic stalemate in ES‐SCLC, giving patients more options [9]. Additionally, the new standard for first‐line treatment for ES‐SCLC is ICIs with chemotherapy.

However, with the increase in immunotherapy research, the potential differences between the safety and efficacy of PD‐1 inhibitors and PD‐L1 inhibitors have raised concerns. For example, Duan et al. published the NMA that mirrored and paired the characteristics of the clinical trials, after which they screened the randomized clinical trials (RCTs) of PD‐1 inhibitors and PD‐L1 inhibitors [10]. Research results demonstrated that, although they were relatively safe, PD‐1 inhibitors were superior to PD‐L1 inhibitors in terms of survival in patients with solid tumors treated generally, either in combination or monotherapy. Similarly, in the first‐line treatment of ES‐SCLC, the comparison of efficacy and safety between PD‐1 inhibitors combined with chemotherapy and PD‐L1 inhibitors combined with chemotherapy is also controversial, and considering that direct head‐to‐head comparisons of PD‐1 inhibitors with chemotherapy and PD‐L1 inhibitors with chemotherapy RCTs in the first‐line treatment of ES‐SCLC are highly improbable, a number of systematic reviews and NMA were conducted by indirect comparison. For example, the NMA by Yu et al. found that for ES‐SCLC, PD‐L1 inhibitors + chemotherapy and PD‐1 inhibitors + chemotherapy had similar efficacy and safety profiles [11]. However, Yang et al.’s meta‐analysis of a retrospective comparative cohort research revealed that patients receiving first‐line PD‐1 inhibitors had longer PFS [12]. The NMA results of Zhou et al. showed that the best first‐line treatment option for SCLC patients may be the combination of PD‐L1 inhibitors (atezolizumab and durvalumab) plus etoposide‐based chemotherapy [13].

In addition, the previous NMA did not include the two recently updated RCTs (CAPSTONE‐1, ASTRUM‐005) [14, 15]. As a result, to the best of our knowledge, this is currently the most comprehensive NMA evaluating the efficacy and safety of PD‐1 inhibitors plus chemotherapy versus PD‐L1 inhibitors plus chemotherapy in the first‐line treatment of ES‐SCLC. And we also conducted some subgroup analyses to figure out if other factors contributed to efficacy for the purpose to minimize potential bias in OS, PFS, ORR, and toxicity, determining the best treatment alternatives in clinical practice and providing thorough evidence to assist both clinicians and patients in making treatment decisions.

## 2 Methods

### 2.1 Protocol and Guidance

All processes for this systematic review and NMA are listed in a prospectively available online registry program (PROSPERO CRD42022363652), which follows the guidelines of PRISMA.

### 2.2 Search Strategy

We searched PubMed, Embase, and the Cochrane Library for all SCLC‐related RCTs from inception to September 2022, with no starting data limits. Additionally, we manually searched the reference lists of every original study, review, and conference presentation from significant international cancer conferences that were available. The search was performed using the following keywords: “small cell lung cancer,” “chemotherapy,” “atezolizumab,” “durvalumab,” “pembrolizumab,” “PD‐1 inhibitors,” “PD‐L1 inhibitors,” “nivolumab,” “serplulimab,” and others. The RCTs used only English as the language of instruction. Two authors (Wenjing Liu and Lulin Yu) performed the search independently, and any differences were discussed with each other to reach a consensus.

### 2.3 Eligibility Criteria

Papers meeting the following criteria were included according to the PICOS framework: (i) Patients diagnosed as ES‐SCLC by histology or cytology were selected; (ii) first‐line treatment studies included the PD‐L1 inhibitors + chemotherapy/PD‐1 inhibitors + chemotherapy cohort and the placebo + chemotherapy cohort of ES‐SCLC patients; (iii) outcomes of the study include the primary endpoints of the research which are the HR and 95% CI for OS and PFS, and the secondary endpoints are the odds risk (OR) and 95% CI for ORR and AEs; (iv) studies that were all Phase 2 or Phase 3 RCTs.

The criteria for exclusion are as follows: (i) single‐arm trials, retrospective studies, phase I clinical trials, observational studies, reviews, and meta‐analyses; (ii) PD‐L1 inhibitors + chemotherapy/PD‐1 inhibitors + chemotherapy cohort as a second‐line and second‐line treatment option for ES‐SCLC patients; (iii) insufficient data for statistical analysis; (iv) when duplicate publications of the same study are discovered, we only include the most recent or most complete studies.

### 2.4 Study Selection

Examine the qualifications of all retrieved studies. If the title and abstract do not provide enough information, we will collect comprehensive publications or conference abstracts. If this approach is not accessible, we will contact the study's author. Choose the most recent publication for studies with more than one publication or trials with overlapping patients. Any doubts are investigated by another auditor and resolved through discussion. No study was omitted due to flaws in the research design or quality.

### 2.5 Data Extraction

To assess eligibility for inclusion in the publication and collect data, two authors (Wenjing Liu and Lulin Yu) independently checked titles, abstracts, full text, and supplementary material according to Cochrane Collaboration requirements. The following details were collected from each study and put into a standard table: (i) essential study information includes study title, first author, year of publication, and patient characteristics. (ii) We extracted HR and 95% CI for OS and PFS, as well as OR and 95% CI for ORR and AEs. (iii) If data for these categories were not reported in the RCT, we treated them as NA (not applicable). (v) To collect the most comprehensive and up‐to‐date data, original tests, supplementary materials, and conference proceeding data information were also assessed.

### 2.6 Risk Assessment of Bias

Two researchers (Siyu Huang and Yuxin Hua) assessed the quality of the included literature using Cochrane bias risk assessment tools, which included randomization, allocation hiding, participant and professional blinding, outcome assessment blinding, inadequate outcome data, selective reporting, and other biases. The outcomes were classified as low risk, high risk, and unclear risk. All differences in quality evaluation are resolved through discussion to reach a consensus among all researchers.

### 2.7 Statistical Analysis

For OS and PFS, HR was chosen as the effect size, and OR was used for ORR and AEs. The inverse variance approach was used to pool the HR and OR. The heterogeneity test uses *I* ^2^ and *p* values to assess study heterogeneity. If *p* > 0.1 and *I* ^2^ ≤ 50%, there is no statistical heterogeneity among the included studies, and the fixed effect model is used for meta‐analysis [16]. If *p* ≤ 0.1 and *I* ^2^ > 50%, it indicates that there is heterogeneity among studies, which should be analyzed and evaluated, and the random effect model should be used for meta‐analysis. There is no statistically significant difference when the 95% CI of the indirect comparison is one. The adjusted indirect comparison used group C (placebo + chemotherapy) as the common treatment group. This NMA indirectly assessed the efficacy and safety of group A (PD‐1 inhibitors + chemotherapy) versus group B (PD‐L1 inhibitors + chemotherapy) by comparing group A versus group C and group B versus group C.

The estimated log HR values were calculated using the equation log HRAB = log HRAC ‐ log HRBC. The standard error (SE) is calculated using the formula: SE (log HRAB) = √SE (log HRAC)2 + SE (log HRBC)2. The calculation of the RRs and related SEs is identical. The relative treatment effect of the HRs and its 95% CIs were evaluated using Bayesian NMA. All comparison variables (PFS, OS, AEs, and ORR) had treatment effect rankings that suggested likelihood. Three Markov chains, each with a distinct starting value, were run concurrently for 50 000 iterations, with refinement intervals of 10 and 20 000 aging for each result. The best probability ranking table is used to rank the outcomes of three intervention options. Subgroup analysis was performed based on ECOG, PD‐L1 expression, liver metastases, and brain metastases in the entire cohort. We collected data from all relevant subgroups included in the trials based on the prearranged subgroup factors. All statistical analyses were performed using R (version 4.0.3) and RStudio software.

## 3 Results

### 3.1 Identification and Selection

**FIGURE 1:**
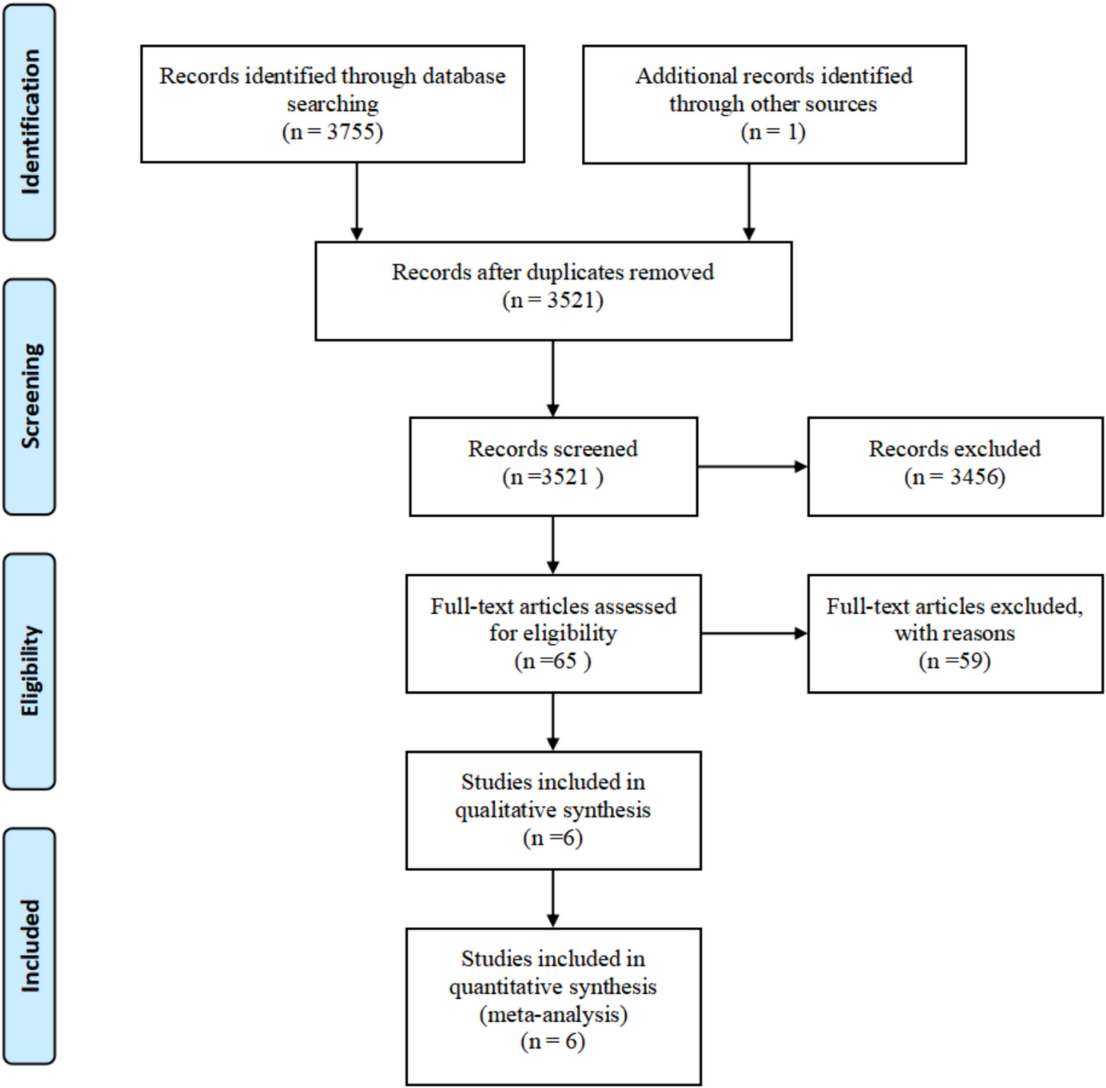
Articles retrieved and assessed for eligibility. After the screening process, six RCT articles met the inclusion criteria and were included in the ultimate analysis.

We initially searched 3756 relevant publications, one of which was a conference report from the 2020 American Society of Clinical Oncology (ASCO), and subsequently, 235 publications were removed due to duplication and 3521 articles were screened. After scanning the titles and abstracts, 3456 articles were omitted, including 39 systematic reviews, 84 reports, and 34 meta‐analyses. Following a thorough examination of the full text, 65 articles were determined to be eligible, with 59 studies ruled out due to single‐arm studies, failure to meet qualifying conditions, and other factors. Finally, this NMA included six RCTs. Figure 1 depicts the search flowchart.

### 3.2 Characteristics of Included Studies and Patients

The study included 3163 participants with a median follow‐up of 12–25 months. Six RCTs (IMpower133, CASPIAN, KEYNOTE‐604, CAPSTONE‐1, ASTRUM‐005, and EA5161) were included, three of these studies explored the efficacy of PD‐L1 inhibitors + chemotherapy (durvalumab, atezolizumab) versus placebo + chemotherapy, while the other three explored PD‐1 inhibitors + chemotherapy (pembrolizumab, serplulimab, and nivolumab) versus placebo + chemotherapy [14, 15, 17, 18, 19, 20]. The primary characteristics of the selected RCTs and their outcomes are displayed in Table 1 and Table S1.

**TABLE 1:** Characteristics of patients and outcomes of included trials.

Trial	Author, year	Phase	Masking	Median follow‐up, months	OR (95% CI) for ORR	HR (95% CI) for OS	HR (95% CI) for PFS	Median age, years	No. of patients	mOS, months	mPFS, months
IMpower‐133	Horn 2018	III	Double‐blind	13.9	NA	0.70 (0.54, 0.91)	0.77 (0.62, 0.96)	64	201	12.30	5.20
								64	202	10.30	4.30
	Stephen 2021	III	Double‐blind	22.9	NA	0.76 (0.60, 0.95)	NA	64	201	12.30	NA
								64	202	10.30	NA
CASPIAN	Paz‐Ares 2019	III	Open‐label	14.2	1.64 (1.12, 2.44)	0.73 (0.59, 0.91)	0.78 (0.65, 0.94)	62	268	13.00	5.10
								63	269	10.30	5.40
	Goldman 2021	III	Open‐label	25.1	1.53 (1.08, 2.18)	0.75 (0.62, 0.91)	0.80 (0.66, 0.96)	62	268	12.90	5.10
								63	269	10.50	5.40
CAPSTONE‐1	Wang 2022	III	Double‐blind	13.5	NA	0.72 (0.58, 0.90)	0.67 (0.54, 0.83)	62	230	15.30	5.80
								62	232	12.80	5.60
KEYNOTE‐604	Charles 2020	III	Double‐blind	21.6	8.9 (0.2, 17.4)	0.80 (0.64, 0.98)	0.75 (0.61, 0.91)	64	228	10.80	4.50
								65	225	9.70	4.30
ASTRUM‐005	Ying 2022	III	Double‐blind	12.3	NA	0.63 (0.49, 0.82)	0.48 (0.38, 0.59)	63	389	15.40	5.50
								62	196	10.90	4.30
EA5161	Leal 2020	II	Open‐label	NA	NA	0.67 (0.46, 0.98)	0.68 (0.48, 1.00)	NA	80	11.30	5.50
								NA	80	8.50	4.70

Abbreviations: HR: hazard ratio; mOS: median overall survival; mPFS: median progression‐free survival; NA: not applicable; OR: odds ratios; ORR: objective response rate; OS: overall survival; PFS: progression‐free survival.

### 3.3 Quality Assessment

Two researchers (Siyu Huang and Yuxin Hua) evaluated the risk of bias for the six RCTs that were included. The risk of bias assessment showed that the risks were within acceptable limits. Randomized sequences for all trials were generated via interactive voice‐response or web‐response system and were therefore assessed as “low risk” for selection bias. With the exception of Paz‐Ares 2019, Spigel 2021, and Leal 2020, all trials were double‐blinded and details of the double‐blind methodology were provided for participants and personnel; therefore, Paz‐Ares 2019, Spigel 2021, and Leal 2020 were rated in terms of implementation bias and detection bias as “unclear risk.” The remainder were rated as “low risk.” The risk of bias summary and risk of bias graph are shown in Figure S1 and Figure S2.

### 3.4 Efficacy

#### 3.4.1 Direct Comparison of PD‐1 Inhibitors + Chemotherapy and PD‐L1 Inhibitors+ Chemotherapy Versus Placebo + Chemotherapy

**FIGURE 2:**
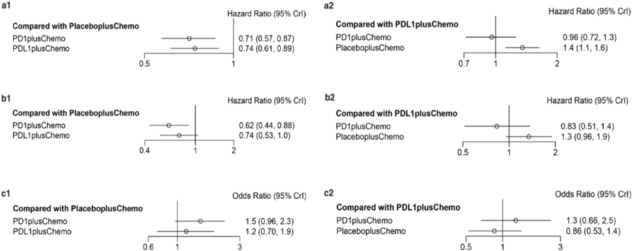
Direct and indirect comparisons between PD‐L1 inhibitors + chemotherapy or PD‐1 inhibitors + chemotherapy with placebo + chemotherapy. Figures a1, b1, and c1 showed the forest plot of HRs and ORs directly comparing OS, PFS, and ORR between PD‐L1 inhibitors + chemotherapy or PD‐1 inhibitors + chemotherapy with placebo + chemotherapy. Figures a2, b2, and c2 showed the forest plot of HRs and ORs indirectly comparing OS, PFS, and ORR between PD‐L1 inhibitors + chemotherapy or PD‐1 inhibitors + chemotherapy with placebo + chemotherapy. Abbreviations: PD‐L1, programmed cell death‐ligand 1; PD‐1, programmed cell death 1; Chemo, chemotherapy; HRs, hazard ratios; ORs, odds ratios; CI, confidence interval.

In direct comparison, as seen in Figure 2(a1, b1), patients receiving PD‐1 inhibitors + chemotherapy exhibited significantly longer OS (HR: 0.71, 95% CI: 0.57–0.87) and PFS (HR:0.62, 0.44–0.88) than patients treated with placebo + chemotherapy. Relative to placebo + chemotherapy, OS (HR: 0.74, 0.61–0.89) was observed with PD‐L1 inhibitors + chemotherapy showing similar improvement as PD‐1 inhibitors + chemotherapy longer than placebo + chemotherapy. However, no significant difference was found in PFS (HR:0.74, 0.53–1.0) observed with PD‐L1 inhibitors + chemotherapy compared to placebo + chemotherapy. Additionally, Figure 2(c1) demonstrates that adding PD‐L1 inhibitors (OR: 1.2, 0.70–1.9) or PD‐1 inhibitors (OR: 1.5, 0.96–2.3) to chemotherapy had not a significant effect on ORR.

#### 3.4.2 Indirect Comparison for OS

**FIGURE 3:**
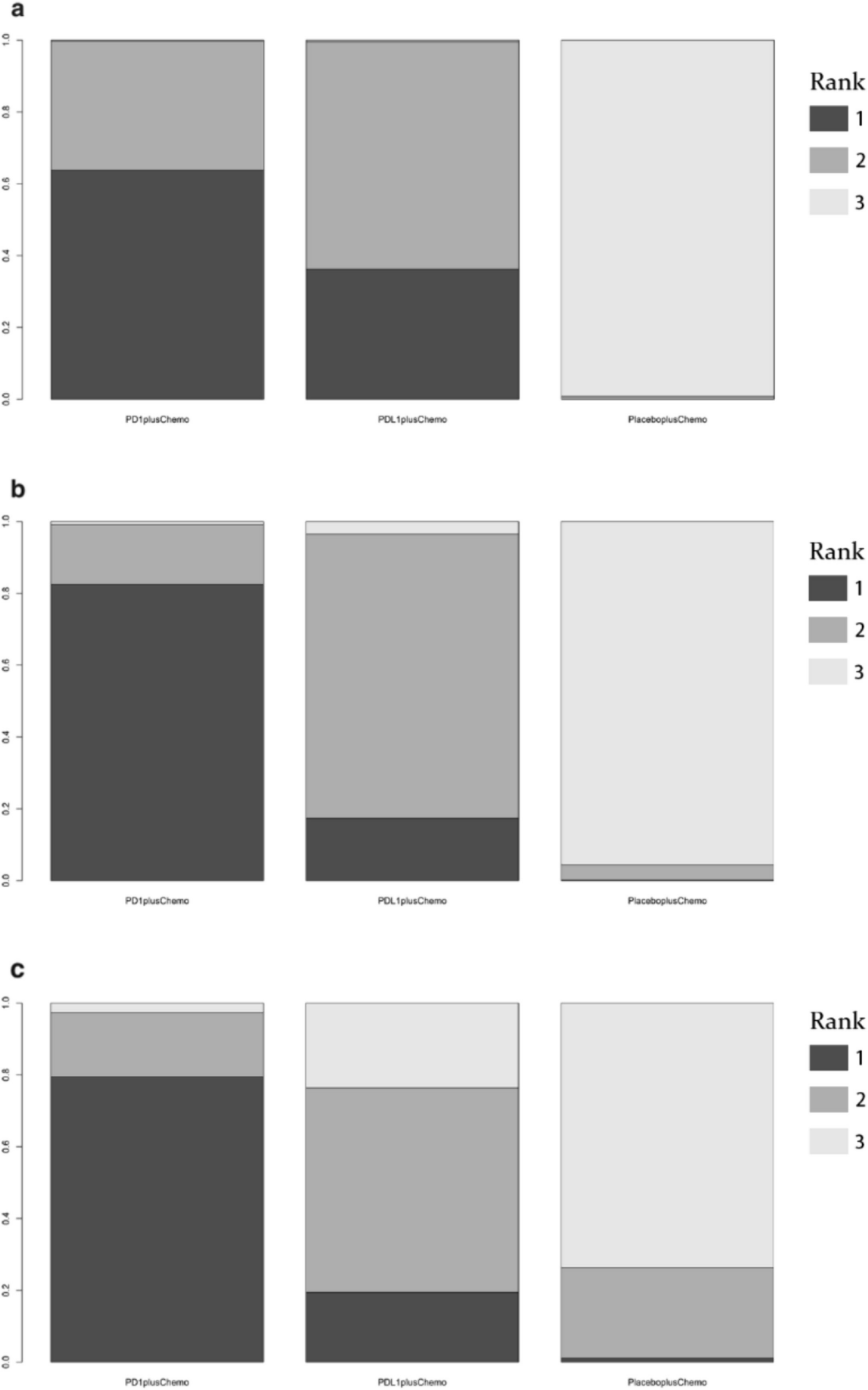
Ranking probabilities base on the multiple comparisons on OS, PFS, and ORR in network meta‐analysis. Ranking probabilities on OS (a), PFS (b), and ORR (c) in ES‐SCLC patients between PD‐L1 inhibitors + chemotherapy or PD‐1 inhibitors + chemotherapy with placebo + chemotherapy.

As shown in Figure 2(a2), there was no significant difference in OS with PD‐1 inhibitors + chemotherapy versus PD‐L1 inhibitors + chemotherapy (HR: 0.96 95% CI: 0.72–1.3), but the Bayesian ranking results revealed that patients receiving PD‐1 inhibitors + chemotherapy had a longer OS than those receiving PD‐L1 inhibitors + chemotherapy (Figure 3a).

#### 3.4.3 Indirect Comparison for PFS

Figure 2(b2) shows that there was no significant distinction in PFS comparing PD‐1 inhibitors + chemotherapy with PD‐L1 inhibitors + chemotherapy (HR 0.83, 95% CI: 0.51–1.4). When the Bayesian ranking data were combined, PD‐1 inhibitors + chemotherapy had the greatest rate of being ranked first (Figure 3b). This finding demonstrates that PD‐1 inhibitors + chemotherapy may be preferable to PD‐L1 inhibitors + chemotherapy and placebo + chemotherapy.

#### 3.4.4 Indirect Comparison for ORR

Figure 2(c2) illustrates that there was no apparent distinction in response generation between patients treated with PD‐L1 inhibitors + chemotherapy and PD‐1 inhibitors + chemotherapy, with the ORR for PD‐L1 inhibitors + chemotherapy versus PD‐1 inhibitors + chemotherapy (OR 1.3 95% CI: 0.66–2.5). Rectangular graphs in the Bayesian ranking show the possibility that each treatment would be ranked from top to bottom in terms of OR and 95% CI. The most likely treatment to be placed first was PD‐1 inhibitors + chemotherapy (Figure 3c).

### 3.5 Safety

#### 3.5.1 Direct Comparison for AEs

**FIGURE 4:**
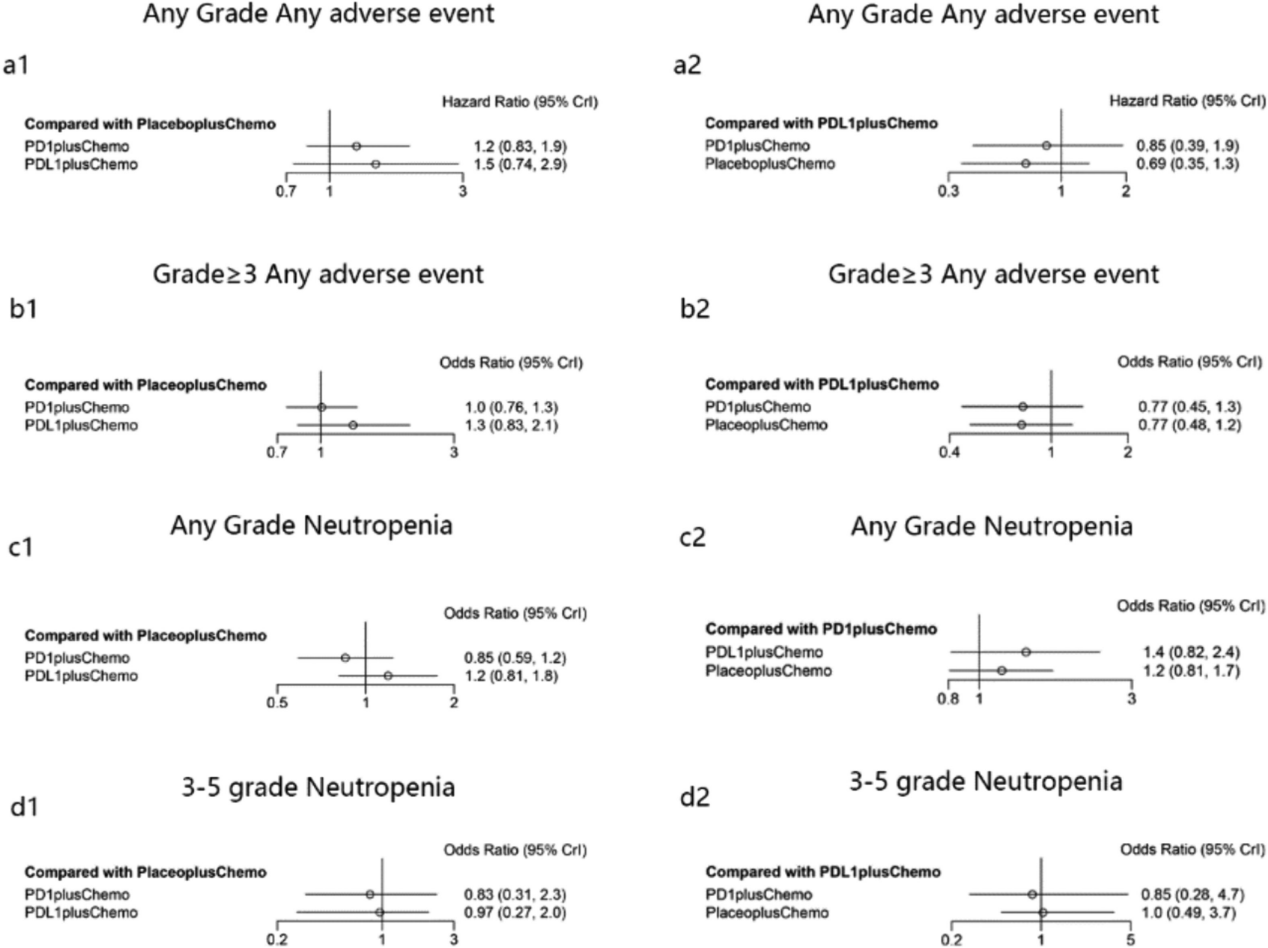
Direct and indirect comparisons of safety among PD‐L1 inhibitors + chemotherapy, PD‐1 inhibitors + chemotherapy, and placebo + chemotherapy. The forest plots of OR for safety comparing PD‐L1 inhibitors + chemotherapy with placebo + chemotherapy and PD‐1 inhibitors + chemotherapy with placebo + chemotherapy were shown in figures a1, b1, c1, and d1, and PD‐L1 inhibitors + chemotherapy with PD‐1 inhibitors + chemotherapy were shown in figures a2, b2, c2, and d2.

In direct comparisons of safety analyses, there were no appreciable differences between PD‐1 inhibitors + chemotherapy or PD‐L1 inhibitors + chemotherapy relative to placebo + chemotherapy with any grade of AE, grade ≥ 3 AE, with any grade of neutropenia, grade ≥ 3 neutropenia (a1, b1, c1, and d1 in Figure 4).

#### 3.5.2 Indirect Comparison for AEs

In indirect comparisons of safety analyses, there were no discernible differences between PD‐L1 inhibitors + chemotherapy relative to PD‐1 inhibitors + chemotherapy in relation to any grade of AE, grade ≥ 3 AE, in relation to any grade of neutropenia, or grade ≥ 3 neutropenia (Figure 4). However, in the Bayesian ranking, the risk of PD‐L1 inhibitors + chemotherapy occurring was least in the occurrence of any grade of AE (a in Figure S3); yet the risk of PD‐L1 inhibitors + chemotherapy occurring was biggest in grade ≥ 3 AE (b in Figure S3), the occurrence of treatment‐related neutropenia of any grade (c in Supplementary Figure 3) and neutropenia of grade 3 or higher (d in Figure S3).

### 3.6 Subgroup Analysis

**FIGURE 5:**
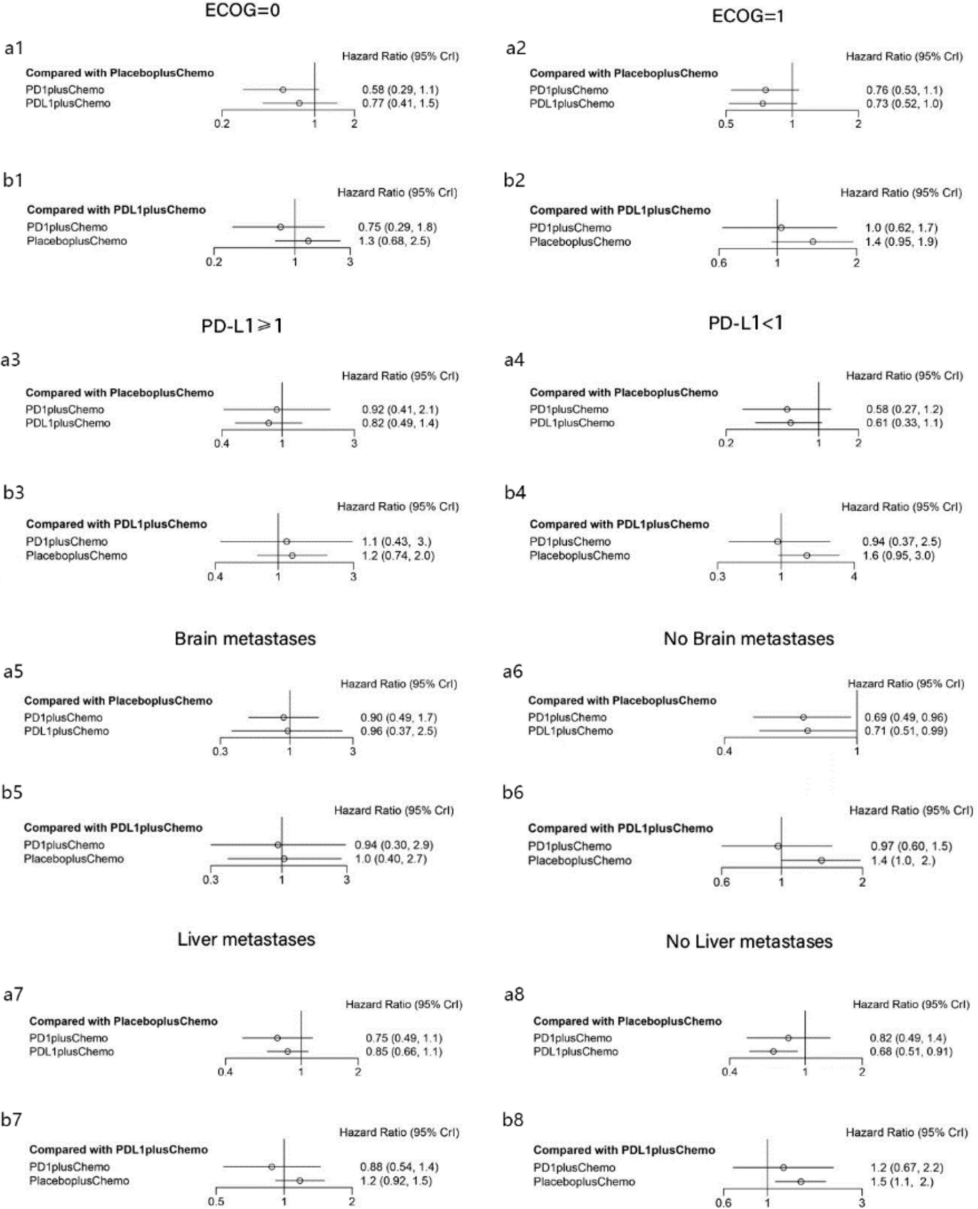
Subgroup analyses for OS in the first‐line treatment of ES‐SCLC. Subgroups including performance status, PD‐L1 expression, liver metastasis status, and brain metastasis status were analyzed. The forest plots of HRs for OS comparing PD‐L1 inhibitors + chemotherapy with placebo + chemotherapy and PD‐1 inhibitors + chemotherapy with placebo + chemotherapy were shown in a1–a8, and PD‐L1 inhibitors + chemotherapy with PD‐1 inhibitors + chemotherapy in b1–b8.

There are a total of three studies with subgroup analysis of OS data: IMpower133, ASTRUM‐005, and KEYNOTE‐604. Figure 5 and Figure S4 display analyses by the presence or absence of liver and brain metastases, PD‐L1 expression, and performance status. Based on baseline liver metastasis status, it showed a significant OS benefit with PD‐1 inhibitors + chemotherapy (HR: 0.68, 0.51–0.91) with placebo + chemotherapy in the subgroup without liver metastasis status, and a significant OS benefit with PD‐L1 inhibitors + chemotherapy (HR: 0.69, 0.49–0.96) with placebo + chemotherapy (a6 and a8 in Figure 5). Besides this, no consistent significant changes in OS were seen in any other special groupings. In the Bayesian ranking (Figure S4), the probability of ranking PD‐L1 in combination with chemo was greatest when ECOG = 1, PD‐L1 expression ≥ 1, with or without brain metastases at baseline, and brain metastasis‐free status at baseline; PD‐1 in combination with chemo was greatest when ECOG = 0, PD‐L1 expression <1, and liver metastasis status at baseline.

### 3.7 Heterogeneity Analysis

Because the number of included articles was less than 10, we could not do publication bias detection. Moreover, given the lack of closed loops in our network diagram, inconsistency assessment was not applicable to our study. Our heterogeneity test showed no significant heterogeneity in OS and any grade of adverse events (a and d in Figure S5), high heterogeneity in PD‐1 inhibitor combination chemotherapy compared with placebo combination chemotherapy in PFS and grade ≥ 3 adverse events (b and e in Figure S5), high heterogeneity in PD‐L1 inhibitor combination chemotherapy compared with placebo combination chemotherapy in ORR(c in Figure S5), and moderate heterogeneity in PD‐L1 inhibitor combination chemotherapy compared with placebo combination chemotherapy in grade ≥ 3 adverse events (e in Figure S5). We speculate that this is because the drugs chosen are different, although they are of the same class; therefore, more clinical studies are needed to confirm our conclusions.

## 4 Discussion

ES‐SCLC, although initially sensitive to chemo, does not have a durable response, and significant treatment resistance is usually observed in recurrent SCLC, with 2‐year survival rates not exceeding 7% [21]. With the development of immunosuppression, PD‐1 and PD‐L1 inhibitors have demonstrated moderate but long‐lasting OS achievements in a subset of patients with SCLC when combined with platinum‐based chemotherapy [22]. However, a key issue of relevance both in the laboratory and in the clinic is the comparison of these agents on the basis of efficacy and toxicity, and we would like to know if there is a difference in therapeutic blockade between PD‐1 inhibitors plus chemotherapy and PD‐L1 inhibitors plus chemotherapy in the first‐line therapy of patients with ES‐SCLC. There are no head‐to‐head randomized controlled trials comparing the efficacy and safety differences between PD‐1 inhibitors plus chemotherapy and PD‐L1 inhibitors plus chemotherapy, and the results of several studies comparing multiple treatments using NMA are controversial, and the two most recently updated RCTs were not included. Therefore, by adjusting for indirect comparisons, we update this NMA to address the distinctions between PD‐1 inhibitors + chemotherapy and PD‐L1 + chemotherapy in the first‐line therapy of ES‐SCLC patients. According to our knowledge, this is the largest NMA of included studies evaluating the effectiveness and security of PD‐1 inhibitors plus chemotherapy and PD‐L1 inhibitors plus chemotherapy in the initial therapy of patients with ES‐SCLC.

Direct comparisons showed that patients who received either PD‐1 inhibitors + chemotherapy (HR: 0.71, 95% CI: 0.57–0.87) or PD‐L1 inhibitors + chemotherapy (HR: 0.74, 0.61–0.89) demonstrated significantly longer OS than those who received placebo + chemotherapy. In terms of PFS, PD‐1 inhibitors + chemotherapy showed similar improvement to placebo + chemotherapy (HR:0.62, 0.44–0.88), while PD‐L1 inhibitors + chemotherapy showed no meaningful difference (HR:0.74, 0.53–1.0).

According to the NMA results, there were no significant distinctions in OS (HR 0.96, 95% CI: 0.72–1.3), PFS (HR 0.83, 95% CI: 0.51–1.4), or ORR (RR 1.3, 95% CI: 0.66–2.5) between PD‐1 inhibitors + chemotherapy and PD‐L1 inhibitors + chemotherapy. However, the Bayesian ranking revealed that patients receiving PD‐1 inhibitors + chemotherapy tended to have longer OS, PFS benefit, and better treatment response than patients receiving PD‐L1 inhibitors + chemotherapy.

In terms of safety, there were no significant differences in either direct or indirect comparisons with any grade of AE, with grade ≥ 3 AE, with any grade of neutropenia, and with grade ≥ neutropenia. However, in Bayesian ranking, the risk of PD‐L1 inhibitors + chemotherapy occurring was least in the occurrence of any grade of AE; yet the risk of PD‐L1 inhibitors + chemotherapy occurring was greatest in grade ≥ 3 AE, with treatment‐related neutropenia of any grade, and neutropenia of grade ≥ 3.

In the subgroup analysis, the probability of PD‐L1 inhibitors + chemotherapy ranking first was greatest when ECOG = 1, PD‐L1 expression ≥ 1, with or without brain metastases at baseline, and with no liver metastasis status at baseline in Bayesian ranking; PD‐1 inhibitors + chemotherapy ranking first was greatest when ECOG = 0, PD‐L1 expression < 1, and with liver metastasis status at baseline. These results provide useful evidence for the need for more individualized treatment for SCLC.

According to a meta‐analysis by Zhou et al., PD‐L1 inhibitors combined with etoposide chemotherapy may be the most effective first‐line therapy for patients with ES‐SCLC [13]. This meta‐analysis of first‐line therapies by patients with ES‐SCLC did not include PD‐1 inhibitors because the literature search was done up until December 2019, when there were no phase II or III clinical trials on PD‐1 inhibitors for first‐line therapies for patients with ES‐SCLC. Instead, it included chemotherapy alone, chemotherapy plus PD‐L1 inhibitors, CTLA‐4 inhibitors, or VEGF inhibitors.

In an indirect comparison meta‐analysis, Yu et al. assessed the effectiveness and safety of PD‐L1 inhibitors + chemotherapy and PD‐1 inhibitors + chemotherapy versus chemotherapy in first‐line therapy of ES‐SCLC. The results of the study revealed that PD‐L1 inhibitors + chemotherapy and PD‐1 inhibitors + chemotherapy significantly improved survival compared to chemotherapy alone [11]. However, PD‐L1 plus chemotherapy and PD‐1 plus chemotherapy had comparable efficacy and safety. This result was validated by the current study, although the included studies were inconsistent, and we added two randomized controlled trials, CAPSTONE‐1 and ASTRUM‐005, However, our findings revealed no significant distinction between PD‐L1 inhibitors + chemotherapy and PD‐1 inhibitors + chemotherapy in terms of efficacy and safety. Yu et al. found that for patients with brain metastases, PD‐L1 inhibitors + chemotherapy had a lower risk of death (HR 0.61; 0.28–1.32), and while our subgroup analysis found no substantial difference between the two methods of therapy, PD‐L1 inhibitors + chemotherapy may be superior to PD‐1 inhibitors + chemotherapy in Bayesian ranking, regardless of the presence of brain metastases at baseline or not.

In contrast to our indirect comparison, Yang et al.’s retrospective cohort‐based meta‐analysis of ES‐SCLC patients treated with PD‐1 inhibitors as first‐line therapy revealed that longer PFS was observed in ES‐SCLC patients treated with PD‐1 inhibitors [12]. But from our Bayesian ranking results, PD‐1 inhibitors + chemotherapy ranked first with the highest probability, a result that suggests that PD‐1 inhibitors + chemotherapy may be superior to PD‐L1 inhibitors + chemotherapy and placebo + chemotherapy. The explanation for this result could be that it was a retrospective study with a small number of patients receiving PD‐L1 inhibitors, and a lack of follow‐up bias and data heterogeneity may have influenced the accuracy of the results to a certain extent. Another factor is that PD‐1 inhibitors have the potential to prevent PD‐1 from binding to both PD‐L1 and PD‐L2, whereas PD‐L1 inhibitors can only prevent PD‐1 from binding to PD‐L1 [23, 24]. Consequently, when given PD‐L1 inhibitors, tumors may circumvent antitumor immune responses via the PD‐1/PD‐L2 axis. Moreover, Chen et al. found that exosomes of malignant melanoma also express PD‐L1. Additionally, these PD‐L1‐expressing exosomes move throughout the body with the blood and have the ability to interact directly with T cells' PD‐L1 monoclonal antibodies, which would reduce the efficacy of PD‐L1 monoclonal antibody [23].

However, more research using accurate animal models is still required to determine the potential mechanisms underlying the variations among PD‐L1 inhibitors and PD‐1 inhibitors in SCLC. Assessment of predictive biomarkers for PD‐L1 inhibitors and PD‐1 inhibitors in SCLC in conjunction with chemotherapy is still fraught with difficulties. The most researched prospective markers for molecular biomarkers are tumor mutational load (TMB) and PD‐L1 expression. These molecular biomarkers can be used to evaluate patients with non‐SCLC (NSCLC) for immunotherapy. For instance, in the first‐line therapy of advanced driver‐negative NSCLC, patients with PD‐L1 ≥ 50% can get a single dose of pembrolizumab [25]. In contrast to NSCLC, PD‐L1 immunohistochemical expression does not predict the outcomes of first‐line chemotherapy in ES‐SCLC [18, 19, 26, 27]. This can be explained in several different ways, and the first is that PD‐L1 seems to be primarily expressed on immune cells and expressed at low levels across the board in SCLC [19, 20, 26, 27]. Secondly, biopsies from SCLC are usually small and mainly consist of necrotic areas [11]. Third, pathology‐based immune cell PD‐L1 scoring has repeatedly shown poor reproducibility in SCLC, thus restricting PD‐L1’s utility as a biomarker in SCLC [28, 29]. Fourthly, PD‐L1 detection reagents and platforms are also diverse to date, with pembrolizumab and nivolumab using Dako22C3 and Dako28‐2 PD‐L1 detection reagents respectively, and atezolizumab and durvalumab using SP263 detection antibodies, and consistency studies of detection antibodies and platforms urgently need to be conducted [30]. Therefore, the use of PD‐L1 as a biomarker still faces many challenges, and there are no criteria for determining a positive cut‐off value for PD‐L1 expression. For TMB, the value of screening a superior population was demonstrated in the CheckMate032 study. Atezolizumab's exploration of TMB did not reach the same conclusion, and the IMpower133 study, the first phase III study to analyze blood‐based TMB (bTMB) as a biomarker for predicting immunotherapy efficacy, did not show an association between bTMB and efficacy [26, 31]. The sample size of the available studies is very limited, and there is no standardization of assay methods, detection platforms, and cut‐off values; therefore, more prospective studies with large samples are needed to support the use of TMB as a predictive biomarker of efficacy.

## 5 Limitations and Perspectives

The following restrictions apply to this study. First, due to a lack of data on individual patients, we were unable to offer subgroup analysis by classifying patients by gender and smoking status, factors which may be related to treatment outcomes. These clinical factors should be taken into account in future research. Second, instead of using data from individual patients, we based our meta‐analysis on published results. Third, due to a lack of data on immune‐related adverse events associated with ICIs and chemotherapy combinations, we were only able to examine the most common events. Fourth, there is a lack of head‐to‐head comparable evidence from randomized controlled studies. Finally, we extracted data from subgroup analyses of RCTs for each treatment. In their subgroup analyses, some studies did not publish OS, HRs, or 95% CIs for OS.

## 6 Conclusion

Overall, our NMA comprehensively compared the efficacy and safety of PD‐1 inhibitors + chemotherapy and PD‐L1 inhibitors + chemotherapy in the first‐line therapy of patients with ES‐SCLC and found no statistically significant differences in terms of OS, PFS, ORR, and AEs in ES‐SCLC based on the limited data available. Due to the lack of direct evidence from RCTs, this study used indirect comparison as an alternative method. In the future, head‐to‐head research can directly compare and support our research results.

## Author Contributions

All authors directly participated in the study and have reviewed and approved the final manuscript. The specific division of labor is as follows: the biostatistician: Kai Zhang; conceptualization: Shanming Ruan, Zhang Kai, and Wenjing Liu; data curation: Wenjing Liu and Lulin Yu; formal analysis: Feng Yuqian; methodology: Zhang Kai and Wenjing Liu; project administration: Wenjing Liu, Lulin Yu, and Feng Yuqian; writing–original draft: Wenjing Liu; writing–review and editing: Wenjing Liu, Lulin Yu, Feng Yuqian, Huang Siyu, Hua Yuxin, and Peng Mingying.

## Ethics Statement

The authors have nothing to report.

## Conflicts of Interest

The authors declare no conflicts of interest.

## Supporting information


**Table S1** Further characteristics of the included trial.
Figure S1 Risk of bias summary.
Figure S2 Risk of bias graph.
Figure S3 Ranking probabilities base on the multiple comparisons on AEs.
Figure S4 Ranking probabilities base on the multiple comparisons on OS in the subgroup analysis.
Method S1 Search strategy for PubMed.
Method S2 Search strategy for Cochrane.
Method S3 Search strategy for Embase.

## Data Availability

The original contributions presented in the study are included in the article/Supplementary Material. Further inquiries can be directed to the corresponding author.
