# Supplementary material for: Which Is More Suitable for First‐Line Treatment of Extensive‐Stage Small Cell Lung Cancer, PD‐L1 Inhibitors Versus PD‐1 Inhibitors? A Systematic Review and Network Meta‐Analysis
Source: Clin Respir J. 2024 Jul 29;18(7):e13804. doi: 10.1111/crj.13804 (PMC11284309; doi:10.1111/crj.13804)

**SUPPLEMENTARY MATERIAL**

**Which is more suitable for** **first-line treatment of extensive-stage small cell lung cancer, PD‑L1 inhibitors versus PD‑1 inhibitors? A Systematic Review and Network Meta-Analysis**

**Supplementary Method**

**Supplementary Method 1** - Search strategy for PubMed

**Supplementary Method 2** - Search strategy for Cochrane

**Supplementary Method 3** - Search strategy for Embase

**Supplementary Table**

**Supplementary Table 1** - Further Characteristics of the included trial

**Supplementary Figure**

**Supplementary Figure 1** - Risk of bias summary

**Supplementary Figure 2** - Risk of bias graph

**Supplementary Figure 3** - Ranking probabilities base on the multiple comparisons on AEs

**Supplementary Figure 4** - Ranking probabilities base on the multiple comparisons on OS in the subgroup analysis.

**Supplementary Method 1** - Search strategy for PubMed

PubMed:423 results

((("Small cell lung cancer"[Title/Abstract] OR "SCLC"[Title/Abstract]) NOT (" Non small cell lung cancer"[Title/Abstract] OR "NSCLC"[Title/Abstract])) AND ((((((((((((("pembrolizumab"[Title/Abstract] OR "nivolumab"[Title/Abstract]) OR "atezolizumab"[Title/Abstract]) OR "durvalumab"[Title/Abstract]) OR "tremelimumab"[Title/Abstract]) OR "avelumab"[Title/Abstract]) OR "immune checkpoint inhibitor"[Title/Abstract]) OR "immune therapy"[Title/Abstract]) OR "immunotherapy"[Title/Abstract]) OR "programmed cell death protein 1"[Title/Abstract]) OR "programmed cell death ligand 1"[Title/Abstract]) OR "PD-1"[Title/Abstract]) OR "PD-L1"[Title/Abstract])) AND (("randomized"[Title/Abstract] OR "trial"[Title/Abstract])OR "clinical"[Title/Abstract])

**Supplementary Method 2** - Search strategy for Cochrane

Cochrane: 3227 results

#1 MeSH descriptor: [Small Cell Lung Carcinoma] explode all trees

#2 “lung” AND ("Small Cell" OR "Small-Cell" )

#3 "pembrolizumab" or "nivolumab" or "atezolizumab" or "durvalumab" or "tremelimumab"or "avelumab" or "immune checkpoint inhibitor" or "immunotherapy" or "programmed cell death ligand 1" or "programmed cell death protein 1" or "PD-1" or "PD-L1"

# 4 (#1 OR #2) AND #3

**Supplementary Method 3** - Search strategy for Embase

Embase:105 results

(‘pembrolizumab’:ab,ti OR ‘nivolumab’:ab,ti OR ‘atezolizumab’:ab,ti OR ‘durvalumab’:ab,ti OR ‘tremelimumab’:ab,ti OR ‘avelumab’:ab,ti OR ‘immune checkpoint inhibitor’:ab,ti OR ‘immunotherapy’:ab,ti OR ‘programmed cell death ligand 1’:ab,ti OR ‘programmed death 1 receptor’:ab,ti OR ‘PD-1’:ab,ti OR ‘PD-L1’:ab,ti) AND (‘randomized controlled trial’/exp))

**Supplementary Table 1** - Further Characteristics of included trials

| **Trial** | **Author, year** | **Stage** | **Line** | **No. of Patients** | **No. of Male** | **No. of Female** | **Group** | **Regimen** | **Check Point** | **ORR(%)** |
| --- | --- | --- | --- | --- | --- | --- | --- | --- | --- | --- |
| IMpower133 | Horn 2018 | extensive-stage | 1 | 403 | 261 | 142 | Intervention Arm | Atezolizumab (1200 mg lV, day 1)+carboplatin (AUC 5 mg/ml/min IV, day 1)+etoposide (100 mg/m² lV, days 1-3) | PD-L1 | 60.2 |
|  |  |  |  |  |  |  | Control Arm | Placebo+ carboplatin (AUC 5 mg/ml/min lV, day 1)+ etoposide (100 mg/m² lV, days 1-3) |  | 64.4 |
|  | Stephen 2021 | extensive-stage | 1 | 403 | 261 | 142 | Intervention Arm | Atezolizumab (1200 mg lV, day 1)+carboplatin (AUC 5 mg/ml/min IV, day 1)+etoposide (100 mg/m² lV, days 1-3) | PD-L1 | NA |
|  |  |  |  |  |  |  | Control Arm | Placebo+ carboplatin (AUC 5 mg/ml/min lV, day 1)+ etoposide (100 mg/m² lV, days 1-3) |  | NA |
| CASPIAN | Paz-Ares 2019 | extensive-stage | 1 | 531 | 374 | 157 | Intervention Arm | Durvalumab (1500 mg IV)+etoposide (80-100 mg/m²) + carboplatin (AUC 5-6 mg/ml/min IV ) / cisplatin (75-80 mg/m² IV ) | PD-L1 | 79 |
|  |  |  |  |  |  |  | Control Arm | Etoposide (80-100 mg/m²) + carboplatin (AUC 5-6 mg/ml/min IV ) / cisplatin (75-80 mg/m² IV ) |  | 70 |
|  | Goldman 2021 | extensive-stage | 1 | 531 | 374 | 157 | Intervention Arm | Durvalumab (1500 mg IV)+etoposide (80-100 mg/m²) + carboplatin (AUC 5-6 mg/ml/min IV ) / cisplatin (75-80 mg/m² IV ) | PD-L1 | 68 |
| CAPSTONE-1 | Wang 2022 | extensive-stage | 1 | 462 | 372 | 90 | Intervention Arm | Adebrelimab (20 mg/kg lV, day 1)+carboplatin (AUC 5 mg/ml/min IV, day 1)+etoposide (100 mg/m² lV, days 1-3) | PD-L1 | 70.4 |
|  |  |  |  |  |  |  | Control Arm | Placebo+ carboplatin (AUC 5 mg/ml/min lV, day 1)+ etoposide (100 mg/m² lV, days 1-3) |  | 69.5 |
| KEYNOTE-604 | Charles 2020 | extensive-stage | 1 | 453 | 294 | 159 | Intervention Arm | Pembrolizumab (200 mg lV)+carboplatin (AUC 5 mg/ml/min IV, day 1) / cisplatin(75 mg/m² lV, days 1)+etoposide (100 mg/m² lV, days 1-3) | PD-1 | 70.6 |
|  |  |  |  |  |  |  | Control Arm | Placebo+ carboplatin (AUC 5 mg/ml/min IV, day 1) / cisplatin(75 mg/m² lV, days 1)+etoposide (100 mg/m² lV, days 1-3) |  | 61.8 |
| ASTRUM-005 | Ying 2022 | extensive-stage | 1 | 585 | 481 | 104 | Intervention Arm | Serplulimab(4.5 mg/kg IV)+carboplatin (AUC 5 mg/ml/min IV, day 1)+etoposide (100 mg/m² lV, days 1-3) | PD-1 | 80.2 |
|  |  |  |  |  |  |  | Control Arm | Placebo+ carboplatin (AUC 5 mg/ml/min lV, day 1)+ etoposide (100 mg/m² lV, days 1-3) |  | 70.4 |
| EA5161 | Leal 2020 | extensive-stage | 1 | 160 | NA | NA | Intervention Arm | Nivolumab (240 mg IV)+Etoposide + carboplatin / cisplatin | PD-1 | 52.29 |
|  |  |  |  |  |  |  | Control Arm | Etoposide + carboplatin / cisplatin |  | 47.71 |

*Abbreviations: ORR Objective response rate;* *NA: Not applicable.*

**Supplementary Figure 1** - Risk of bias summary: Review authors’ judgments about each risk of bias item for each included study.


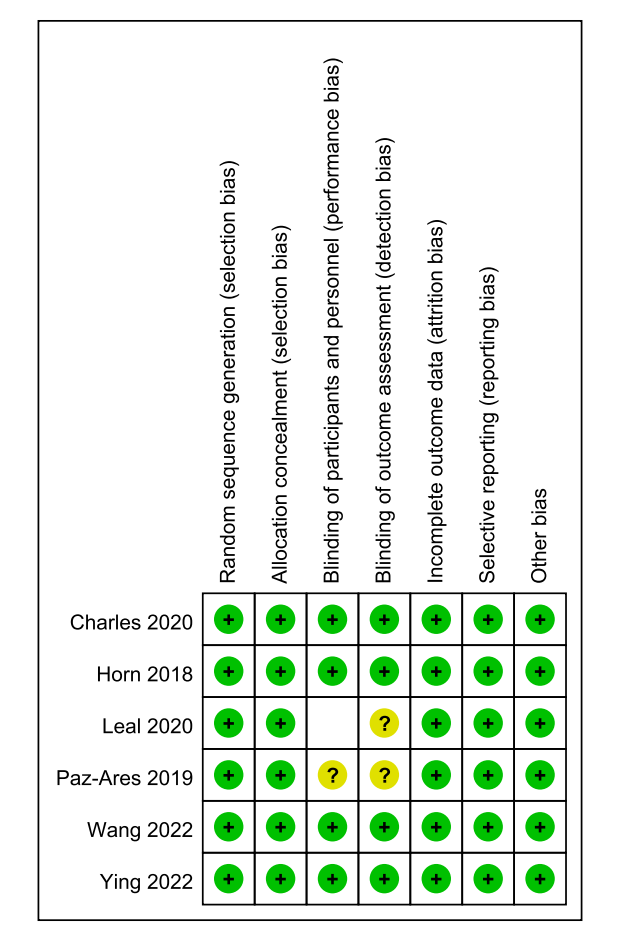


**Supplementary Figure 2** - Risk of bias graph: Review authors’ judgments about each risk of bias item for each included study.


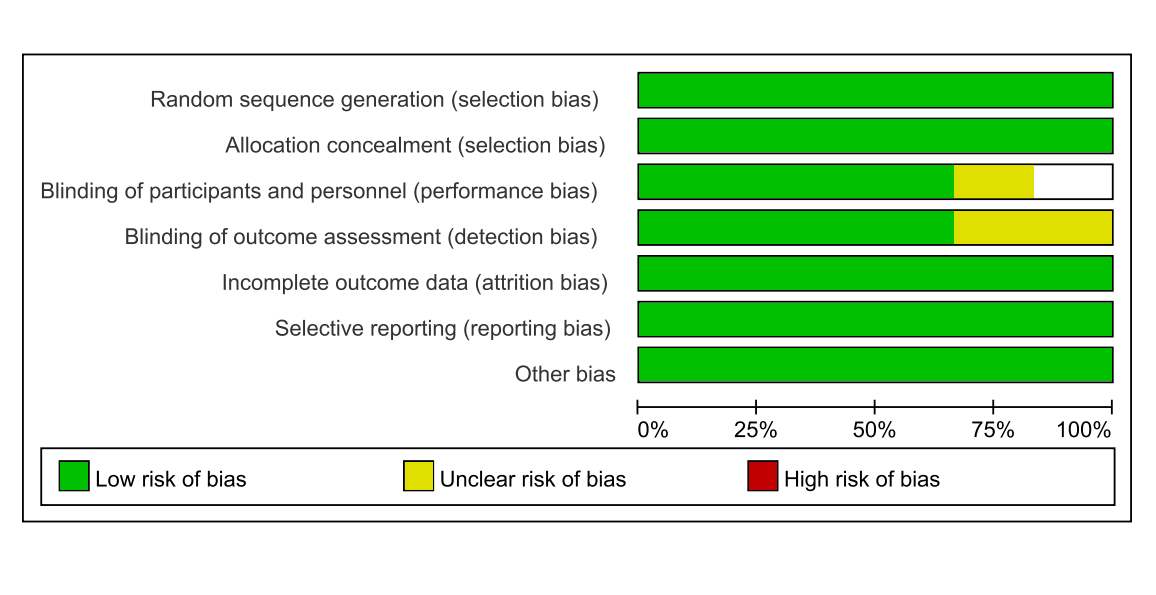


**Supplementary Figure 3 -** Ranking probabilities base on the multiple comparisons on adverse in the analysis.Including any grade any adverse event(a), grade≥3 adverse event(b), any grade neutropenia(c) and 3-5 grade neutropenia(d).

**
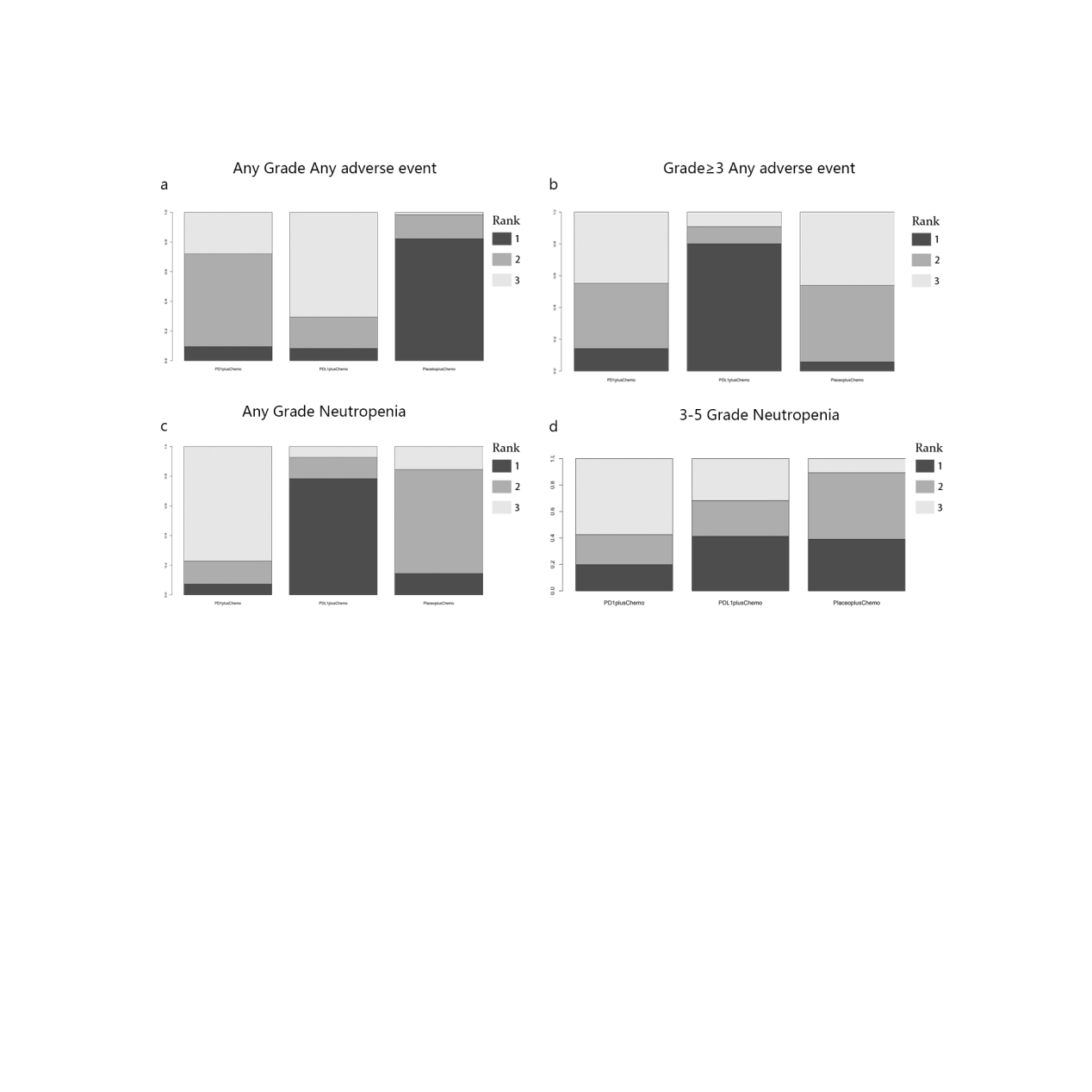
**

**Supplementary Figure 4 -** Ranking probabilities base on the multiple comparisons on OS in the subgroup analysis. Subgroups including performance status(a, b), PD-L1 expression(c, d)，liver metastasis status(e, f), and brain metastasis status(g, h) in ES-SCLC patients between PD-L1 + Chemo or PD-1 + Chemo with Placebo + Chemo were analyzed.


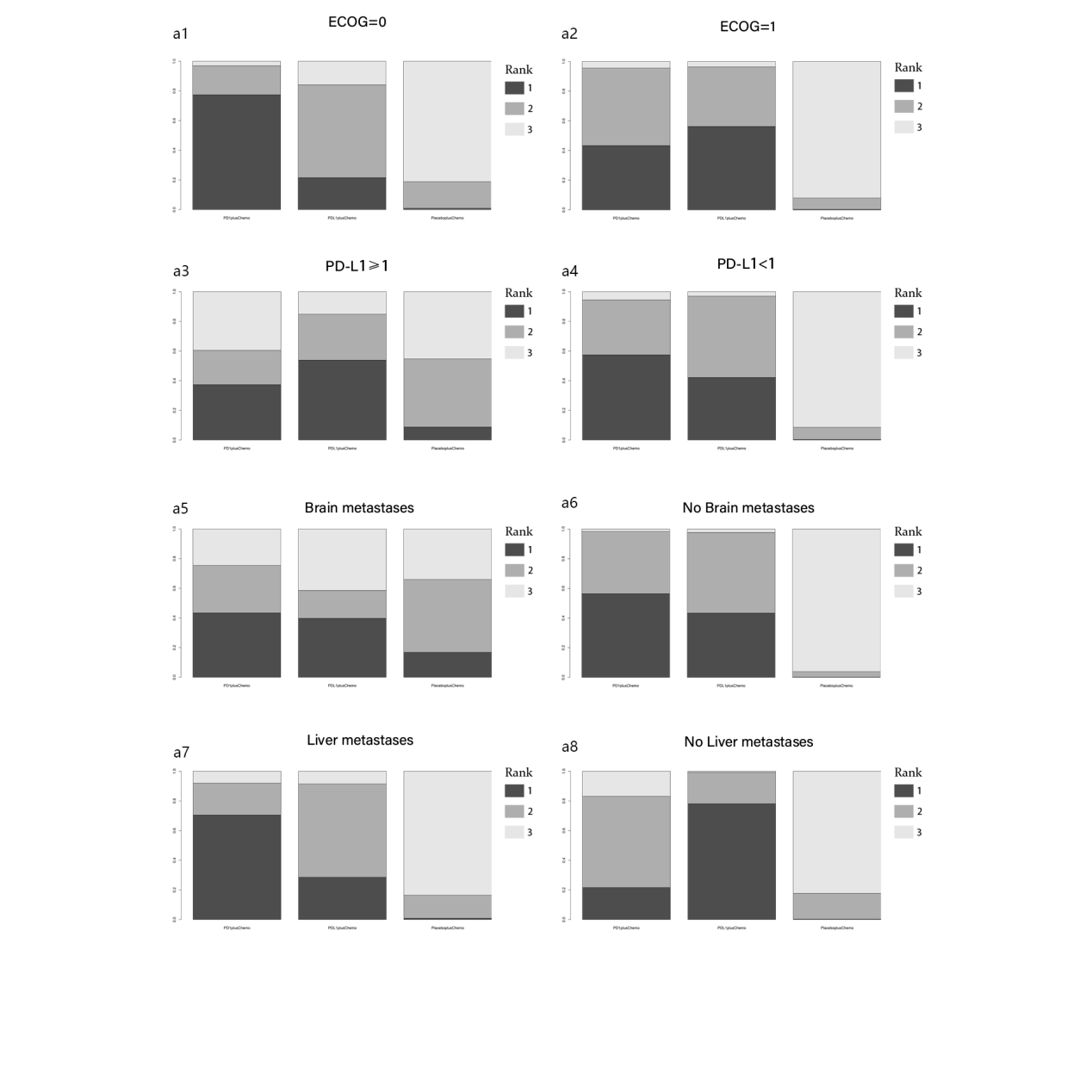

Supplement: Supplementary file 1 — Table S1 Further characteristics of the included trial. Figure S1 Risk of bias summary. Figure S2 Risk of bias graph. Figure S3 Ranking probabilities base on the multiple comparisons on AEs. Figure S4 Ranking probabilities base on the multiple comparisons on OS in the subgroup analysis. Method S1 Search strategy for PubMed. Method S2 Search strategy for Cochrane. Method S3 Search strategy for Embase. [file CRJ-18-e13804-s001.docx]
